# The Use of Vertiflex® Interspinous Spacer Device in Patients With Lumbar Spinal Stenosis and Concurrent Medical Comorbidities

**DOI:** 10.7759/cureus.5374

**Published:** 2019-08-12

**Authors:** Jason Hartman, Michelle Granville, Robert E Jacobson

**Affiliations:** 1 Pain Medicine, Spine and Orthopedic Center, Santa Barbara, USA; 2 Neurological Surgery, University of Miami Hospital, Miami, USA

**Keywords:** vertiflex interspinous implants, lumbar spinal stenosis, neurogenic claudication, coflex interlaminar implant, lateral recess stenosis, lumbar degenerative spondylolisthesis

## Abstract

The use of the Vertiflex® interspinous spacer is a recent minimal invasive procedure useful in the treatment of lumbar spinal stenosis (LSS). It is used mostly by interventional pain physicians who can also perform the minimally invasive lumbar decompression (MILD procedure). Previously when a patient had clinical symptomatic neurogenic claudication (NC) and radiologic findings of lumbar stenosis and had failed conservative treatment, the options were decompressive laminectomy, laminectomy with pedicle fixation at one or more levels or laminotomy combined with interlaminar stabilization (Coflex® implant). These procedures were performed by neurosurgeons and orthopedic spine surgeons. However, the majority of patients with LSS are elderly and have multiple comorbidities that can make open spinal surgery, even when limited to one level, an anesthesia risk as well as vulnerable to the risk associated with hospitalization and recovery after spine surgery. The minimally invasive approaches to interspinous stabilization make it possible to treat localized symptomatic stenosis in a broader group of patients that do not want or cannot, have general anesthesia or extensive lumbar surgery, especially in the prone position. This article examines the use of the Vertiflex® implant in an elderly population with significant comorbidities that underwent successful outpatient implantation at one or two levels. In addition, it serves to familiarize spine surgeons about the possibility of using more minimal approaches to treat LSS.

## Introduction

The use of the minimally invasive Vertiflex® (Vertiflex Inc., Carlsbad, USA) interspinous spacer for the treatment of symptomatic lumbar spinal stenosis (LSS) provides another surgical option in the treatment of neurogenic claudication. Studies have shown the use of the Vertiflex® implant to be effective in both short term and five-year follow-up studies in relieving neurogenic claudication for localized symptomatic LSS [1]. Similar to other minimally invasive techniques it has specific advantages over more open spinal surgical procedures, including shorter procedure time, the possibility to be performed under local anesthesia, minimal or no muscle disruption or blood loss and less risks of nerve damage or cerebrospinal fluid leaks [2]. In this paper, we review its use as an option for more elderly patients and those with medical issues and comorbidities that would preclude the use of both general anesthesia and more open and extensive surgical procedures such as laminectomy with pedicle screw fixation and Coflex® interlaminar implants (Paradigm Spine, New York, USA) after limited decompressive laminotomy but where the patients have significant functional restriction secondary to worsening symptoms of neurogenic claudication. In this paper, a group of Vertiflex patients was analyzed from a larger group of patients that were treated within the same neurosurgical practice that was able to perform the entire range of open and minimally invasive procedures for LSS. Patients were selected for implantation of the Vertiflex® based on a combination of patient symptoms, localized radiologic findings, patient preference or comorbidities that were regarded as contraindication to more extensive open procedures.

## Materials and methods

Patient charts from a single neurosurgical practice that underwent various surgical procedures for symptomatic lumbar stenosis, including multilevel laminectomy with or without pedicle screw fixation, as well as Coflex® and Vertiflex® implantation were retrospectively reviewed.* *This review then looked at the comorbidities of the Vertiflex patients. 

After correlating history, physical exam and radiologic studies, the risk factors reviewed were the patient's age, body mass index (BMI), smoking status, diabetes, cardiac and pulmonary disease, anti-coagulant use, and osteoporosis. 

All patients had an evaluation of the clinical and functional severity of their low back pain and neurogenic claudication was then assessed using the pain Visual Analogue Scale (VAS), Oswestry Disability Index (ODI), and Zurich Claudication Questionnaire (ZCQ). Thirteen patients underwent Vertiflex® for symptoms of intermittent neurogenic claudication, including pain, discomfort, heaviness, cramping or numbness that radiates to the buttock, thigh and lower leg after walking for a certain distance. Detailed diagnostic imaging evaluation was performed including lumbar x-rays, with flexion and extension and sagittal and axial image evaluations with both MRI and CT scans.

Based on the accepted criteria for using the Vertiflex® implant, including symptomatic neurogenic claudication (NC) with radiologic confirmed LSS of 50% or less confined to one or two levels, Vertiflex® was used because of anesthesia concerns and/or significant restrictions secondary to comorbidities [1]. 

## Results

Over 24 months a total of 86 patients underwent various surgical treatments of symptomatic neurogenic claudication for lumbar spinal stenosis. The most common level was L4-5 and the most associated radiologic condition was degenerative stenosis with grade 1 spondylolisthesis. Seventy-three patients underwent open surgery. Forty-five had either laminectomy alone or with pedicle screw fixation, 20 at one level, usually L4-5 and 25 at two or more levels. Limited laminotomy combined with Coflex®^ ^implantation at one or two levels was performed in 28 patients, 20 at one level and eight at two levels. Of the remaining 13 Vertiflex patients, eleven patients had single-level implants, nine at L4-5 and two at L3-4. Ten of the 13 had grade 1 spondylolisthesis, mostly at L4-5. Two patients had two-level implants. All patients had surgery performed under local anesthesia with light sedation, in ambulatory surgery and were discharged home the same day. There were no postoperative wound infections or hematomas. One patient persisted with severe claudication and underwent decompressive laminectomy with pedicle fixation for L4-5 stenosis and degenerative spondylolisthesis. In contrast, patients undergoing pedicle screw fixation were all performed under general anesthesia with an average hospital stay of 2.8 days. There were no wound infections or hematomas but six of the 45 open cases had delayed cerebrospinal fluid (CSF) leaks requiring drainage and prolonged hospitalization and two had repeat surgery to seal the leak. Six developed urinary retention requiring catheterization. Two developed thrombophlebitis. Of the 28 Coflex patients, 15, all one level, were done under general anesthesia by one surgeon, all staying in the hospital for an average of 1.4 days. Eleven had a Coflex® at one or two levels by another surgeon under local anesthesia with sedation in an ambulatory surgery setting and discharged home. Of the 13 outpatient Coflex patients five had two-level implants and eight - one-level. One patient that had an L4-5 Coflex®, 20 months later, had a Vertiflex® implant performed at the adjacent L3-4 level under local anesthesia.

The Vertiflex patients had a Zurich Claudication Index (ZCQ) score that ranged from 54.7% to 75.5%, similar to the other patients' groups subjected to open surgery, indicating that there was a significant effect on the patient's quality of life. 

The medical/anesthesia risk factors included in this assessment were the patient's age, BMI, smoking status, diabetes, cardiac and pulmonary disease, anti-coagulant use, and osteoporosis. In our 13 patients we found that the mean age was 75.46 years old, ranging from 55 to 91 years, with five being 79 or older. The average age of open surgery was similar however there were more patients over 80 years of age in the Vertiflex group. The mean BMI was 30.31 and five patients, had a BMI of 35 or 38%, compared to the open surgical group where very few patients (less than 10%) had a BMI over 35. Thirty-one percent of our patients were either current smokers or previous smokers. When looking at their medical history, 46% were controlled diabetics on medication, 69% had cardiac history and 46% had a history of either asthma or chronic obstructive pulmonary disease (COPD) and 23% had a history of osteoporosis with a prior osteoporotic spinal compression fracture. Only 16% of these patients were on anti-coagulant medications that had to be stopped before the procedure (Table 1).

**Table 1:** Table of comorbidities Table showing age, BMI (basic metabolic index), smoking status, cardiac morbidity, pulmonary morbidity, diabetes, anticoagulant use and other comorbidities HTN: Hypertension ASA: Aspirin A-fib: Atrial Fibrillation COPD: Chronic Obstructive Pulmonary Disease HX: History

Age	BMI	Smoker	Cardiac	Pulmonary	Diabetes	Anticoagulants	Osteoporosis	Vertiflex Levels
91	25	No	No	Asthma	No	No	No	L3-4
71	35.24	No	HTN	Asthma	Yes: type 2	No	No	L4-5
80	29.81	No	Chronic A-fib, HTN	COPD	Yes: type 2	Yes: ASA, Xarelto	No	L4-5
67	36.05	Former	HTN	No	Yes: type 2	No	No	L4-5
71	37.28	No	HTN	No	No	No	No	L3-4
73	36.92	Former	HTN, aortic atherosclerosis	No	Yes with diabetic neuropathy	No	No	L4-5
87	31.04	Former	HTN	No	Yes: type 2	ASA	Yes: hx of fracture	L4-5
77	24.03	No	No	Asthma	No	No	Yes: hx of fracture	L4-5
79	28.07	No	HTN	No	No	No	No	L3-4, L4-5
79	18.89	Yes	No	No	No	No	Yes: hx of fracture	L4-5
55	39.27	No	HTN	Asthma	Yes type 2	No	No	L2-3, L3-4
76	29.52	No	HTN	COPD	No	No	No	L4-5
75	23.56	No	No	No	No	No	No	L4-5

## Discussion

Lumbar spinal stenosis (LSS) is a progressive narrowing of the spinal canal, commonly due to either facet and ligamentous hypertrophy and is often associated with degenerative spondylolisthesis, most commonly at L4-5 [1]. Although many patients are found with radiologic stenosis with no or minimal symptoms, the hallmark symptom is progressive neurogenic claudication relieved by sitting and worsening with standing and walking. Stenosis is radiologically identified most commonly at L4-5 but also can be seen at L3-4 level or multiple levels but without significant motion with flexion and extension films. A common finding on both MRI and CT scan of the lumbar spine in patients with LSS is posterior "crowding" of the spinal canal secondary to hypertrophy and inward buckling of the ligamentum flavum which is worsened with extension [1, 3]. Stenosis is often not a single plane pathology so it is critical to understand that narrowing not only affects the axial area but overall spinal canal volume over the entire length of the stenosis. It is important to evaluate and compare the volume changes between the bone and ligaments and the dural sac over multiple slices on MRI and CT scans [3]. Pain and functional evaluation include VAS and ODI as well as functional testing such as ZCQ scale and distance walking. Symptomatic neurogenic claudication typically shows the ZCQ scores should be worse than the scores for back pain and disability reflected in lower VAS and ODI scores. Relief with sitting and worsening with standing and walking is a classic presentation. Detailed imaging evaluation with an assessment of the severity of neurogenic claudication symptoms is a critical step in determining the procedure that is most appropriate for the patient. Determining if the stenosis is localized to one or two spinal segments or more multilevel and diffuse is important as well as establishing if the spondylolisthesis is mild (5-7 mm or less) if there is motion on flexion and extension films and if the stenosis involves less than 50% of the spinal canal and due to ligamentous hypertrophy rather than bone overgrowth. All these radiologic criteria combined with clinical complaints and ZCQ and ODI scores contribute to the decision regarding surgical options [1, 3-4].

Surgery for spinal stenosis was the fastest-growing type of lumbar surgery in the United States from 1980-2000 and while from 2002-2007 surgical rates overall declined, complex fusion procedures increased 15 fold, from 1.3 to 19.9 per 100,000 beneficiaries [5-6]. With LSS being the most common reason for patients older than 65 years old to have spinal surgery, the assessment of comorbidities, along with the radiographic evaluation of the anatomic severity and degree of neurogenic claudication symptoms, is essential in the decision making as to which procedure is most appropriate for the patient. It is well established that the greater the invasiveness of a procedure the greater the risk of intra-operative complications due to more extensive dissection, decortication of bone, longer operative time, anesthesia risks and possible placement of implants and potential neural damage or cerebrospinal fluid leaks [5-8]. Furthermore, studies confirm that fusion is associated with greater complications and postoperative mortality than decompression alone [6-7]. Extensive lumbar surgical procedures can also carry the consequences of increased healthcare use from longer hospital stays, skilled nursing facility use and revision surgery [8].

Many patients respond to physical therapy and localized facet and epidural blocks, but ultimately patients with worsening neurogenic claudication need to consider surgery. While many options exist for these patients including open surgical procedures such as pedicle screw fixation (PSF) and interbody fusion to a less extensive midline and partial lateral recess decompression with Coflex® midline interlaminar stabilization device combined with the use of the Vertiflex® interspinous spacer for the treatment of LSS offers a minimally invasive, percutaneous option for patients that are at an increased anesthesia and surgical risk due to comorbidities such as increased age, diabetes, cardio-pulmonary pathology, obesity or high body-mass index (BMI), liver and kidney disease and cortico-steroid use to name a few [6-7]. Risk factors for immediate postoperative complications in spine surgeries are multi-factorial. Increased patient age and contaminated or infected wounds were identified as independent predictors of mortality. Increased patient age, cardiac disease, pre-operative neurologic abnormalities, prior wound infection, corticosteroid use, history of sepsis, American Society of Anesthesiologists (ASA) classification of >II, and prolonged operative times were independent predictors for the development of one or more complications [8, 2]. In one study of complex spinal procedures major medical complications were reported in 3.1% of patients overall, and wound complications in 1.2%. Mortality was 0.4% within 30 days of discharge [5]. 

In some patients a combination of comorbidities can exclude more invasive complex procedures entirely, while others can be associated with relative risk factors and it is up to the individual physician and his medical and anesthesia team to assess these factors. Shorter surgical time and minimal incisions allow the use of local anesthesia with or without mild sedation rather than general anesthesia. Although diabetes mellitus (DM) is not a contraindication for more aggressive surgery, patients with diabetes have a significantly higher incidence of chronic kidney disease (CKD), hypertension (HTN), cardiovascular disease (CVD), cerebrovascular disease (CbVD) poorer wound healing than non-DM patients and the prevalence ratio of DM in patients with LSS was 24.3% [9-10]. DM and DM with pathologic sequelae are closely associated with postoperative surgical site infection, leading to a worse prognosis. One study reported that DM is the most important predictor of surgical site infection after lumbar spinal surgery and surgical management of LSS demonstrated a rate of side effects ranging from 10% to 24% in surgical cases [9-10]. With this information, we can extrapolate that while the percutaneous use of Vertiflex® is not immune to these complications due to DM it may be a superior choice in the right patient due to shorter intra-operative time and decreased tissue disruption. The actual timing of surgery, the patient's comorbidities such as age, diabetes, obesity, pulmonary limitations and effects on intra-operative positioning, use of anticoagulants, significant osteoporosis - all play a factor in pre- and perioperative decisions, as well as the choice of anesthesia type and type of surgery [2, 10].

**Figure 1:**
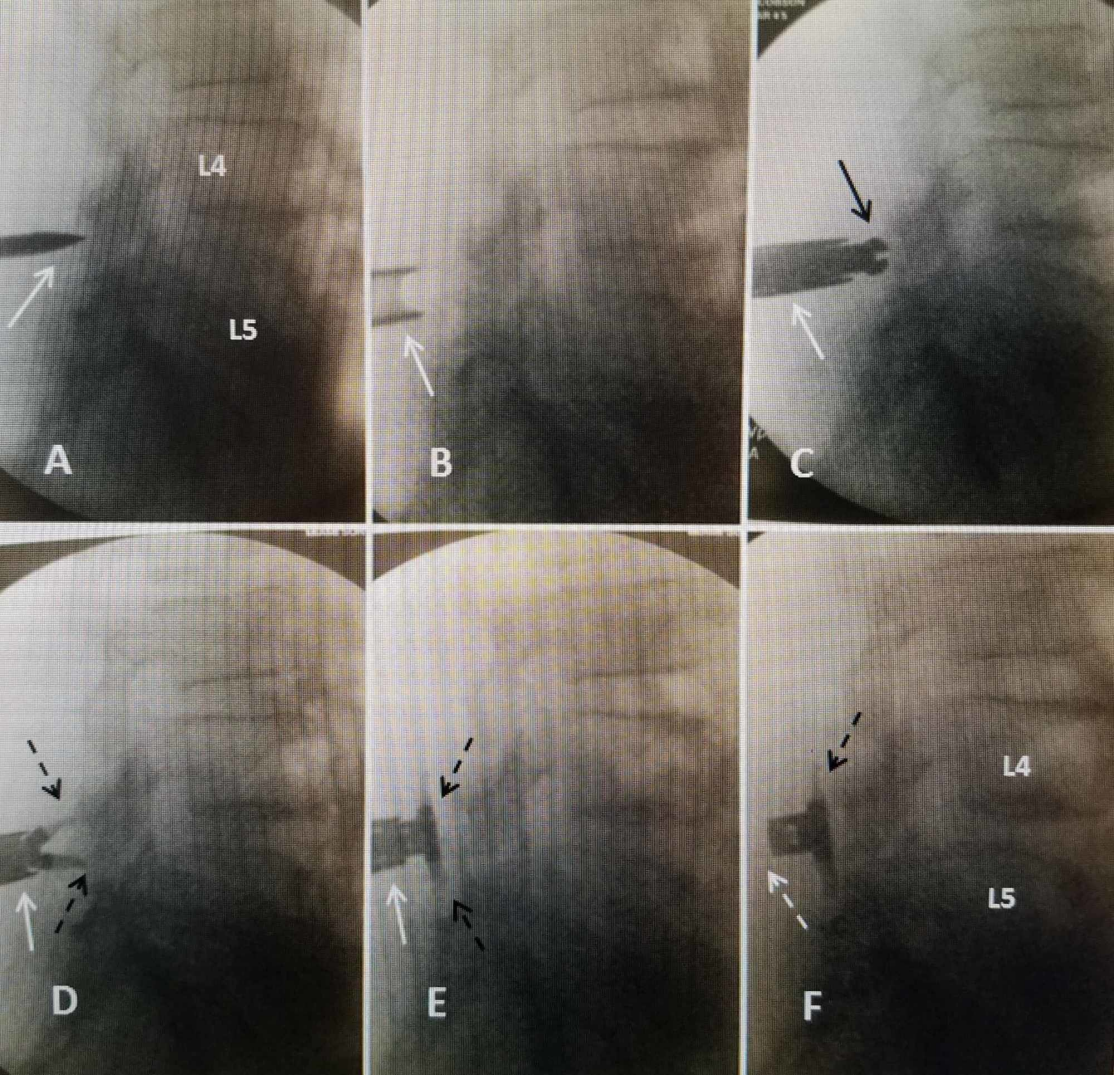
Sequential intra-operative images of insertion of 12 mm Vertiflex® implant for L4-5 stenosis and grade 1 spondylolisthesis. All positions are confirmed both in lateral and anterior-posterior views. A: Insertion of interspinous biconcave curved dissector (solid white arrow) making path through interspinous ligament. B: The outer dilator cannula (solid white arrow) after insertion over the dissector and the dissector has been removed. C:  The "sizer" (solid black arrow) passed down and just distal to the dilator tube (solid white arrow) is partially opened to determine the size of implant ranging from 10 to 16 mm. D: The Vertiflex® implant (dashed black arrow) passed just distal to the cannula in a closed position just before starting to "deploy" in the interspinous space. The Vertiflex® is extended just distal to the insertion tube to allow "wings" to open on both sides of the superior and inferior spinous processes. E: 12 mm Vertiflex® implant fully opened (dashed black arrow) along the interspinous space but still attached to insertion device (solid white arrow). F: Vertiflex® in the L4-5 interspinous space after it is separated from the handle. The wings are deployed (dashed black arrows) and the interspinous body can be seen (dashed white arrow).

**Figure 2:**
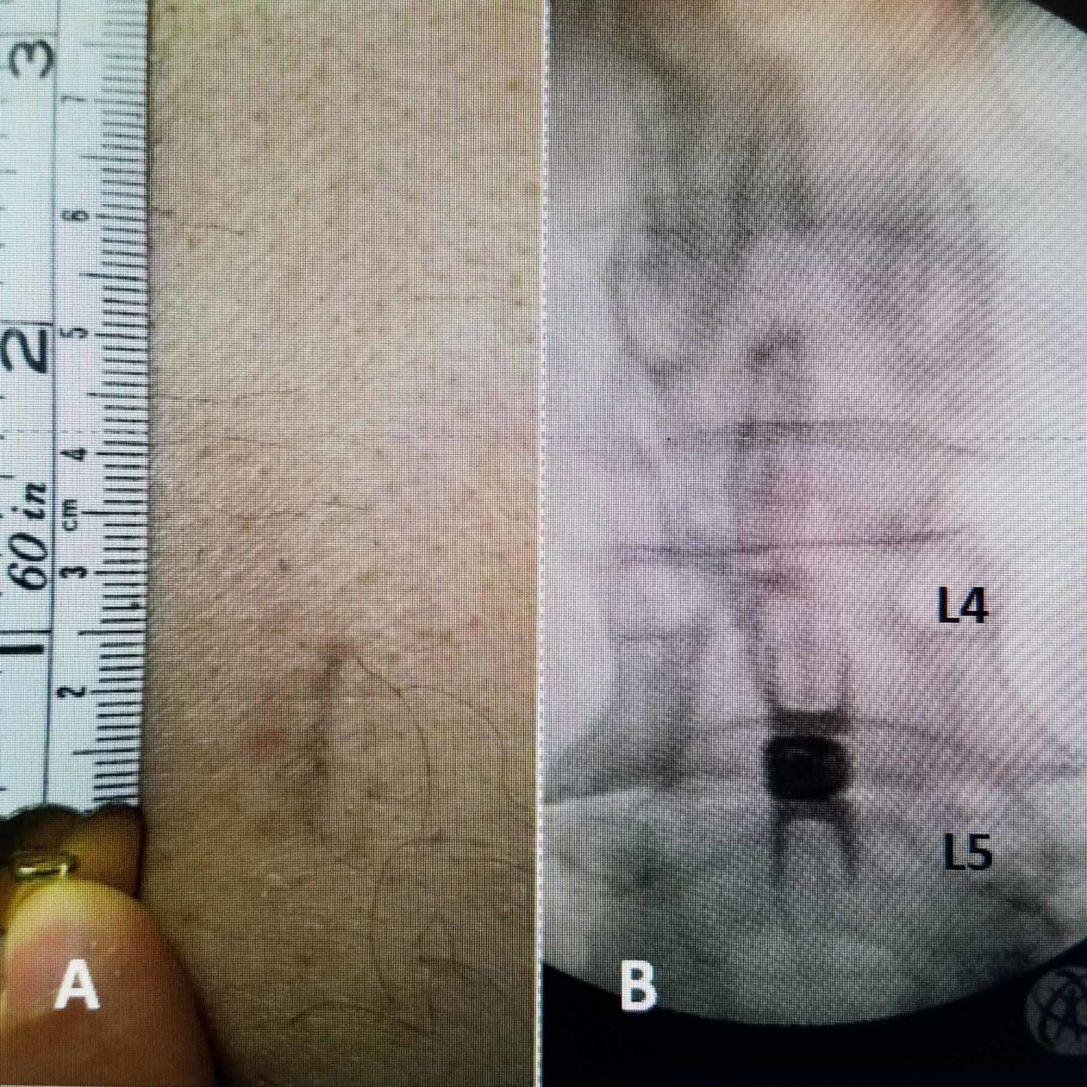
Skin issue of 15 mm for insertion of L4-5 Vertiflex® interspinous implant A: Midline skin incision of 15 mm with no paraspinal dissection to insert the interspinous device. B: 12 mm implant centered over L4-5 interspinous space. Both "deployed" wings superiorly and inferiorly are around the mid spinous processes.

Indications for the Vertiflex® implant include patients suffering from pain, numbness, and/or cramping in the legs (neurogenic intermittent claudication) secondary to a diagnosis of moderate lumbar spinal stenosis, with or without grade 1 spondylolisthesis, confirmed by X-ray, MRI and/or CT evidence of thickened ligamentum flavum, narrowed lateral recess, and/or central canal narrowing [11-12]. The Vertiflex® Interspinous Spacer is indicated for those patients with impaired physical function who experience relief in flexion from symptoms of leg/buttock/groin pain, with or without back pain, who have undergone at least six months of non-operative treatment. The Superion® Interspinous Spacer may be implanted at one or two adjacent lumbar levels in patients in whom operative treatment is indicated at no more than two levels, from L1 to L5 [12]. Contraindications to Vertiflex® are instability of the lumbar spine, e.g., isthmic spondylolisthesis or degenerative spondylolisthesis greater than grade 1, an ankylosed segment at the affected level(s), fracture of the spinous process, pars interarticularis, or laminae (unilateral or bilateral), scoliosis (Cobb angle >10 degrees), cauda equina syndrome, diagnosis of severe osteoporosis, defined as bone mineral density in the spine or hip that is more than -2.5 S.D. below the mean of adult normals, active systemic infection, or infection localized to the site of implantation, prior fusion or decompression procedure at the index level and morbid obesity defined as a body mass index (BMI) greater than 40 [1, 10-12]. Using a minimal percutaneous procedure, like the Vertiflex® implant allows stabilization with mild distraction (Figure 1 and 2).

**Figure 3:**
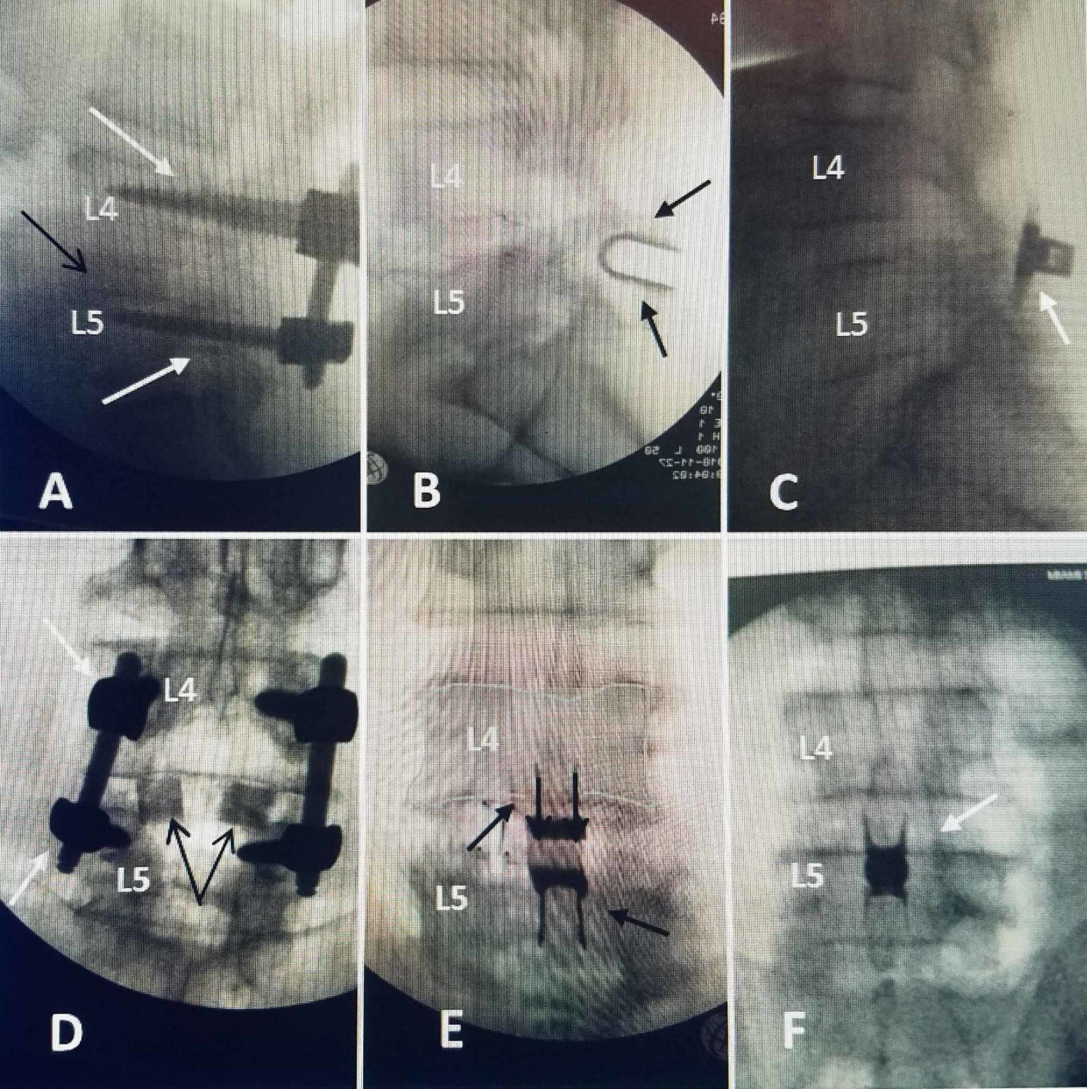
Postoperative radiographs of different L4-5 stenosis cases showing relative size difference between pedicle screws, Coflex® interlaminar and Vertiflex® interspinous implants. The photographs were kept to similar size so the size of the different systems can be compared. A: Lateral postoperative radiograph of one level L4-5 pedicle screw fixation (solid white arrows) with bilateral decompression, medial facetectomy and foraminotomy with interbody grafts (solid black arrow). B: Lateral radiograph of one-level Coflex® implant (solid black arrows) at L4-5. C: Lateral radiograph after L4-5 one-level Veriflex® implant (solid white arrow). D: Anterior-posterior radiograph showing L4 and L5 pedicle screws and rods and two interbody implants in the L4-5 interspace (solid black arrows). E: Anterior-posterior view of L4-5 Coflex® in after interlaminar decompression positioned between the two spinous processes at L4-5 (solid black arrow). F: Anterior-posterior view of Vertiflex® interspinous device (solid white arrow) placed percutaneously.

Another major factor in procedure selection is the surgeon's personal experience, familiarity with interpreting MRI and CT for spinal stenosis, preferences and familiarity with the different procedures [13-14]. Generally the procedures including decompression alone may be sufficient when there is not significant instability or only low grade spondylolisthesis, especially in an elderly, relatively inactive patient. Pedicle screw fixation (PSF) and interbody fusion are used with wider decompression, decompression involving the facet joints or if there is clear instability and movement with flexion and extension radiographs. However, using pedicle screws in elderly osetoporotic patients may have risk of screw loosening as well as dural tears from adherence of the chronic ligamemtous hypertrophy to the dura [5, 8] Another option is limited midline and partial lateral recess decompression combined with interlaminar stabilization using a Coflex® interlaminar device and the Vertiflex® without decompression (Figure 3). 

Different physician specialties have evaluated and treated LSS using different procedures, so it is possible that the selection of a procedure may be often influenced by the preference and specialty of the surgeon, usually a neurosurgeon or orthopedic spine surgeon, rather than clear differences in outcomes. In fact, there are no comparable studies comparing the different procedures when performed by the same surgical groups. During the different FDA approval processes for each device, often many years apart, Coflex® was compared to laminectomy and pedicle screw fixation, while Vertiflex® was compared to other interspinous devices. The more minimal procedures suggest that even the smallest incremental improvement in overall canal area without wide decompression can be effective in providing symptomatic relief in elderly patients [13, 15]. In the experienced hands, minimally invasive lumbar decompression and tubular decompression using various size tubes and instruments to resect the hypertrophied ligament or facet bone without interspinous distraction or stabilization of the spinal segment can also relieve symptomatic neurogenic claudication. The specialty and experience of the physician evaluating the patient plays a large role in the type of procedure recommended or performed [3, 11]. With the introduction and training of interventional pain physicians to evaluate and also perform minimal procedures for stenosis such as radiofrequency ablation, MILD decompression and Vertiflex® interspinous stabilization, it may be valuable to establish an algorithm to help determine the best procedure based on different clinical and radiologic stages of severity of LSS [1, 11, 14-15]. Interventional pain specialists include radiologists, interventional pain physicians and anesthesiologists, many of whom may not be totally familiar with the nuances of clinical and radiologic evaluation of patients with lumbar spinal stenosis and neurogenic claudication. It is equally important they be aware of the various other surgical options offered since there can be a bias in the type of procedure offered to the patient based on the physicians' ability and options. The converse is also true in that neurosurgeons and orthopedic spine surgeons may not be familiar with the more minimal procedures, so they only consider more extensive surgical procedures in patients that could achieve good to excellent relief of their neurogenic claudication with a much simpler procedure. This is especially the case in advanced aged patients who have medical comorbidities that increase the intra-operative and postoperative risks. Considering less invasive procedures will broaden their ability to offer these procedures especially in patients with significant comorbidities or other reasons to offer more minimal approaches that with a proper selection have similar short and long-term outcomes.

## Conclusions

Deciding on the timing and the appropriate surgical treatment option for a patient with symptomatic LSS and neurogenic claudication is determined by many factors. These include radiologic evaluation of the levels and severity of the stenosis, the extent of the clinical symptoms and if they totally resolve with positional changes, such as sitting compared to standing and walking. This article looks at the role of medical comorbidities that may make larger open surgery and general anesthesia higher risk or even contraindicated. The treating physician's specialty and experience with different procedures must also be considered as well as the age, anesthesia risk and comorbidities such as obesity, diabetes and cardio-pulmonary restrictions which may make the option of procedures such as MILD or Vertiflex® reasonable. On the other hand, physicians only trained to perform more minimal procedures must also be able to recognize when the patient is better suited for a more extensive surgical decompression with or without stabilization. 
